# Using the structural diversity of RNA: protein interfaces to selectively target RNA with small molecules in cells: methods and perspectives

**DOI:** 10.3389/fmolb.2023.1298441

**Published:** 2023-11-16

**Authors:** Aixiao Li, Ahmed Bouhss, Marie-Jeanne Clément, Cyril Bauvais, J. Paul Taylor, Guillaume Bollot, David Pastré

**Affiliations:** ^1^ Synsight, Genopole Entreprises, Evry, France; ^2^ Université Paris-Saclay, INSERM U1204, Université d’Évry, Structure-Activité des Biomolécules Normales et Pathologiques (SABNP), Evry, France; ^3^ Department of Cell and Molecular Biology, St. Jude Children’s Research Hospital, Memphis, TN, United States

**Keywords:** targeting RNA: protein interaction, RNA-binding proteins, small molecules, drug screening, cellular assay

## Abstract

In recent years, RNA has gained traction both as a therapeutic molecule and as a therapeutic target in several human pathologies. In this review, we consider the approach of targeting RNA using small molecules for both research and therapeutic purposes. Given the primary challenge presented by the low structural diversity of RNA, we discuss the potential for targeting RNA: protein interactions to enhance the structural and sequence specificity of drug candidates. We review available tools and inherent challenges in this approach, ranging from adapted bioinformatics tools to *in vitro* and cellular high-throughput screening and functional analysis. We further consider two critical steps in targeting RNA/protein interactions: first, the integration of *in silico* and structural analyses to improve the efficacy of molecules by identifying scaffolds with high affinity, and second, increasing the likelihood of identifying on-target compounds in cells through a combination of high-throughput approaches and functional assays. We anticipate that the development of a new class of molecules targeting RNA: protein interactions to prevent physio-pathological mechanisms could significantly expand the arsenal of effective therapeutic compounds.

## 1 Introduction

Although RNA has emerged as a promising therapeutic target for many disease states, the development of small molecules targeting RNA remains in its very early stages (Thomas and Hergenrother, 2008; Donlic and Hargrove, 2018; Warner et al., 2018; Costales et al., 2020; Childs-Disney et al., 2022). Potential applications encompass a wide variety of human pathologies, including neurodegenerative diseases (Korobeynikov et al., 2022), cancers, (Li and Chen, 2013), viral infections, and other rare diseases. RNA targets include both protein-coding mRNAs and non-coding RNAs that regulate the expression of proteins associated with pathologies (e.g., oncogenes). RNA also participates in pathological deregulation of alternative splicing, contributes to pathological intercellular communications, and regulates cell differentiation and apoptosis.

**FIGURE 1:**
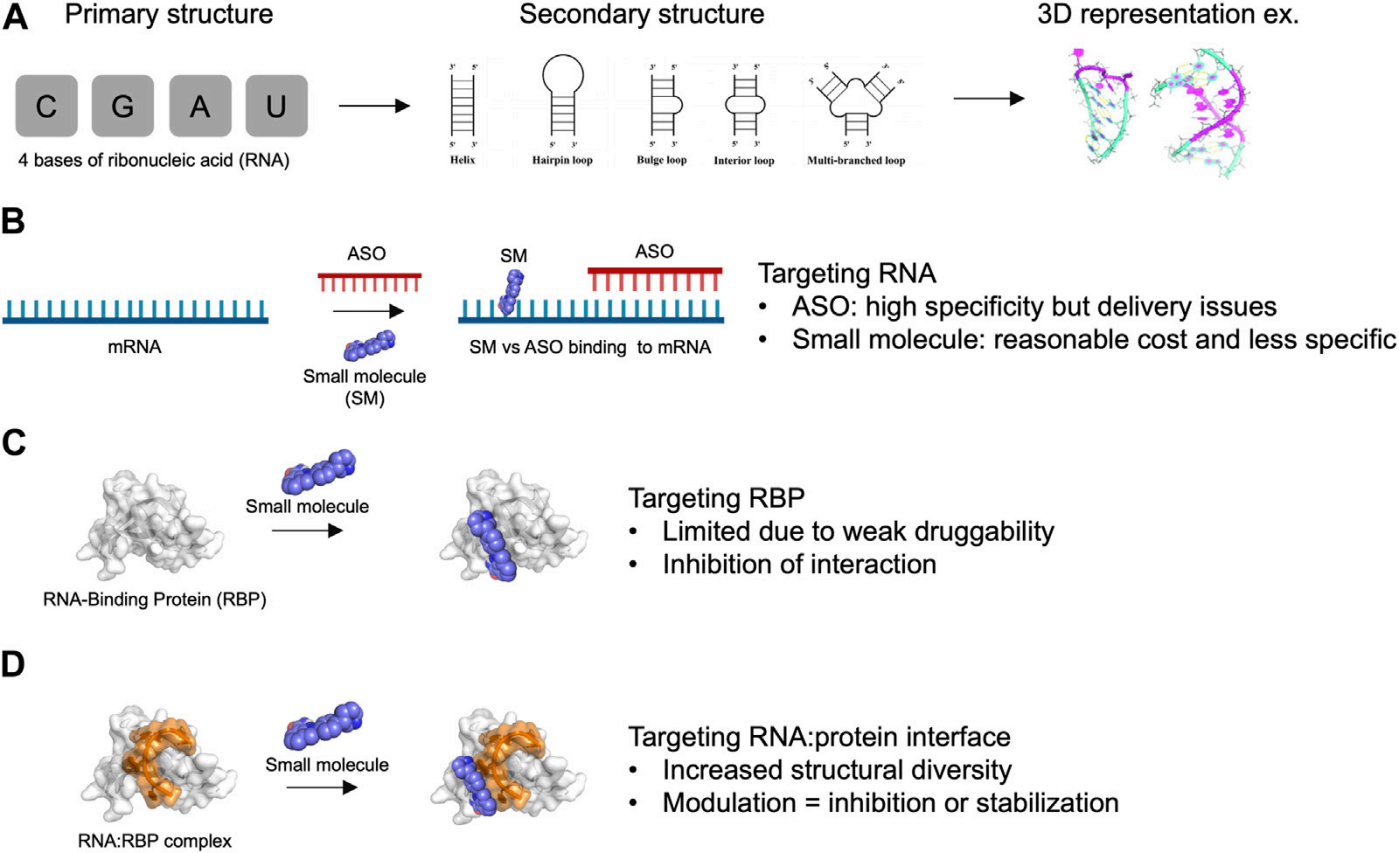
Advantages and limitations of targeting RNA and RNA-Binding Protein with small molecules. **(A)** Primary, secondary structures and 3D representation of RNA composed by only 4 bases of ribonucleic acids. **(B)** Targeting RNA with antisense oligonucleotides (ASO) *versus* small molecules (SM). **(C)** Targeting RNA-Binding Protein (RBP) with small molecules. **(D)** Targeting RNA:RBP interface with small molecules. The association of RNA with protein provide an increased structural diversity that may be useful for drug development.

Various strategies have been pursued for targeting RNA, which we categorize into two groups (Figures 1A, B): 1) strategies that directly target RNA, including antisense oligonucleotides (ASO) (Crooke et al., 2018) and CRISPR gene editing (Gunitseva et al., 2023), and 2) small molecules designed to specifically recognize RNAs of interest. ASOs have yielded promising results in human trials, paving the way for the use of RNA as therapeutic targets (Oren et al., 2022; Tran et al., 2022; Dindot et al., 2023). However, several obstacles remain for the development of such gene therapy, linked to factors such as cost, nucleic acid delivery, toxicity, and side effects (Shen et al., 2019). These challenges collectively hinder the development of gene therapy for most common human diseases in which RNA plays a central role, such as cancer and neurodegeneration.

Small molecules offer significant advantages in terms of affordability, ease of delivery to patients, and potential oral administration (Angelbello et al., 2020). However, small molecules may have limited selectivity towards a specific RNA of interest, primarily due to the low sequence diversity of RNA, which consists of only 4 bases. While RNA molecules can adopt various secondary structures that can be targeted (Childs-Disney et al., 2022), such as stem-loops and bulges, their structural diversity remains relatively limited compared to proteins (Figure 1B). Moreover, small molecules typically interact with only few RNA bases (1–8 at most) that are highly redundant in the human genome (Ursu et al., 2020). Therefore, the development of small molecules targeting specific RNAs presents a significant challenge. In this respect, ASO- and CRISPR-based approaches that target RNAs via direct base-pairing over tens of nucleotides can achieve a level of specificity that has not yet been attained with small molecules.

In this review, we explore the possibility of targeting RNA:protein interfaces to improve the specificity of small molecules to RNAs in cells. Indeed, compared with individual RNA molecules, RNA:protein interfaces (Gerstberger et al., 2014; Hentze et al., 2018; Wu, 2020; Julio and Backus, 2021) have much greater structural diversity due to a combination of independent parameters: 1) the secondary structures adopted by the RNA molecule, 2) the sequence of the RNA bases, 3) the secondary/ternary structures of the protein domain forming (or bearing) the interface pocket, and 4) the nature of amino acid residues involved in the interaction with RNA (Figures 1C, D). However, one notable obstacle is that RNA-binding proteins (RBPs) possess a limited number of structured domains that are present in most of the thousands of RBPs that have been identified in the human genome (Gerstberger et al., 2014). Furthermore, structured domains such as RNA-recognition motifs (RRM) or K homology (KH) domains are considered poorly druggable (Minuesa et al., 2019) since they lack the well-defined hydrophobic pockets that are found in many enzymes and kinases (Figure 1C). In addition, although RBPs have many unstructured domains that participate in the binding of proteins to RNA, their lack of defined structure and the redundancy in their amino acid compositions make them more challenging targets for small molecules compared to structured domains. Molecules designed to interact with low complexity domains of RBPs are the subject of ongoing research (Babinchak et al., 2020; Biesaga et al., 2021) due to the role of RBPs in liquid-liquid phase separation in cells. However, this strategy will not be detailed here. Rather, we focus on RNA:protein complexes for which structural data can be obtained to develop a structure-based strategy for targeting specific RNA: protein complexes.

Below, we critically review current techniques and technologies that have been employed to target RNA, RBPs, and the shared interface between RNA and RBPs. We further propose that integrative approaches will be necessary to identify new molecules targeting RNA: protein interfaces (Schneider et al., 2020; Shaker et al., 2021). First, small molecules must be selected from large libraries and screened experimentally to identify potential molecular hits. This step is critical and can be quite difficult. *In silico* screening based on computer-aided approaches can be used, but must be developed in a rational manner (Childs-Disney et al., 2022; Sadybekov and Katritch, 2023) by using structural data (Zafferani and Hargrove, 2021; Wang et al., 2023). The selection of small molecules requires high-throughput screening using various techniques (Julio and Backus, 2021), including fluorescence polarization or FRET (fluorescence resonance energy transfer), for which advantages and disadvantages are discussed. While *in vitro* approaches are valuable, it is also important to determine whether small molecules are on-target in a cellular context (El Hage et al., 2023). Several parameters, such as membrane crossing, specific folding within cells, and non-specific binding to other biomolecules, cannot be predicted *in vitro*. Functional assays are also crucial to further investigate the efficacy of small molecules. These assays can measure whether translation, splicing, transcription, or other cellular functions controlled by the targeted RNA: protein interaction are modulated in the presence of small molecules. However, as we discuss below, functional assays may be misleading if used in isolation without additional complementary approaches to decipher whether the small molecules are engaging with their intended targets. Finally, we offer our perspectives on developing a new class of RNA:protein inhibitors to treat human pathologies.

## 2 *In silico* approaches to assess the efficacy of small molecules targeting RNA: protein interactions

Drugging RNA with small molecules (Garber, 2023), especially by targeting RNA: protein interactions, requires both classical and innovative *in silico* approaches to assess their effectiveness. In recent years, the use of *in silico* methods, including artificial intelligence (Melo et al., 2022; Staszak et al., 2022), has become essential. These methods enable the prediction of biological activities, analysis of binding parameters, and indications of the physicochemical properties of chemical compounds, ultimately reducing costs and time associated with traditional drug discovery (Jabalia et al., 2021).

### 2.1 Virtual screening algorithms

**FIGURE 2:**
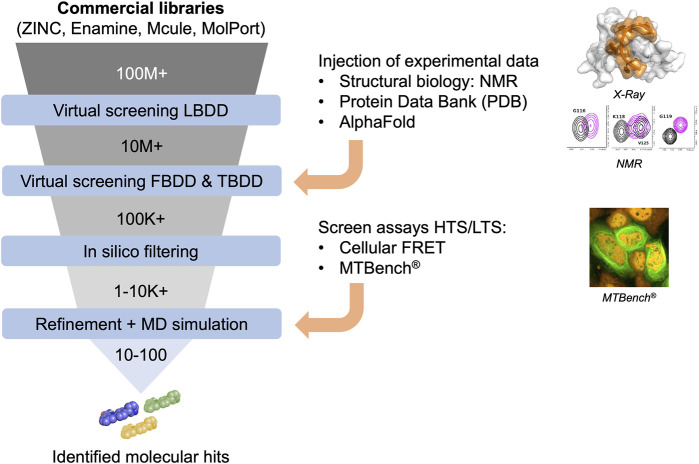
Funnel of *in silico* compound screening based on commercial libraries involving Ligand-, Fragment-, and Target-based approaches improved by experimental data to obtain molecular hits.

*In silico* approaches in drug discovery involve three types of virtual screening methods (Figure 2): ligand-based, target-based, and fragment-based. The ligand-based approach utilizes chemical compounds (e.g., RNA-focused libraries) as references to identify molecules with similar biological activities. The target-based approach relies on 3-dimensional structures of targets (de Almeida Paiva et al., 2022), particularly proteins, to identify compounds that can interact with specific binding sites through virtual screening. Today, several RBP structures are available in apoform or in complex with nucleic acids, providing a robust starting point for applying this approach. Target-based virtual screening has also been applied to resolved RNA secondary structures to identify small molecules (Kallert et al., 2022). Finally, the fragment-based approach aims to test low-molecular-weight molecules and generate candidate compounds from chemical fragments with low structural complexity and high chemical diversity. These approaches offer efficient ways to prioritize and select potential drug candidates in a cost-effective manner, leveraging computational techniques and analysis.

### 2.2 Artificial intelligence in drug discovery

The increasing availability of extensive databases containing molecular information enables the use of machine learning-based predictive models, which rely on substantial data from active and inactive compounds to make accurate predictions (Oliveira et al., 2023). Consequently, machine learning techniques such as deep learning have gained popularity in virtual screening due to their high accuracy, expanded chemical libraries, novel molecular descriptors, and effective similarity search techniques.

For more than 50 years, the world has addressed the problem of predicting RNA secondary structure by using computational methods. More recently, with the increasing availability of RNA structure data, machine learning and especially deep learning algorithms have been developed to tackle this challenging problem and are still in progress (Sato et al., 2021; Zhao et al., 2021; Justyna et al., 2023). Recent work has utilized generative models to design new chemical compounds based on the protein binding pocket topology, offering an improved target-based drug design approach. This approach could be applied to target RNA: protein interfaces and especially the RBP pocket (Shi et al., 2022). In the case of a ligand-based approach, generative models such as reinforcement learning have been developed to create chemical analogs using a scoring function composed of various rewards such as physicochemical properties (Zhou et al., 2019; Korshunova et al., 2022).

Today, several artificial intelligence tools have been developed to revolutionize nearly every stage of the drug discovery process, including target identification (Zhavoronkov et al., 2020), molecular simulations (Noé et al., 2020) and 3D structure prediction such as AlphaFold (Jumper et al., 2021; Varadi et al., 2022), predicting of drug properties (Noé et al., 2020; Paul et al., 2021), *de novo* drug design (Schneider, 2018; Paul et al., 2021), candidate drug prioritization (Li et al., 2020), and synthesis pathway generation (Noé et al., 2020). These tools offer substantial potential to reshape the speed and economics of the pharmaceutical industry, particularly when targeting RNA with small molecules.

## 3 Structural data to validate or screen small molecules targeting RNA: protein interactions

**FIGURE 3:**
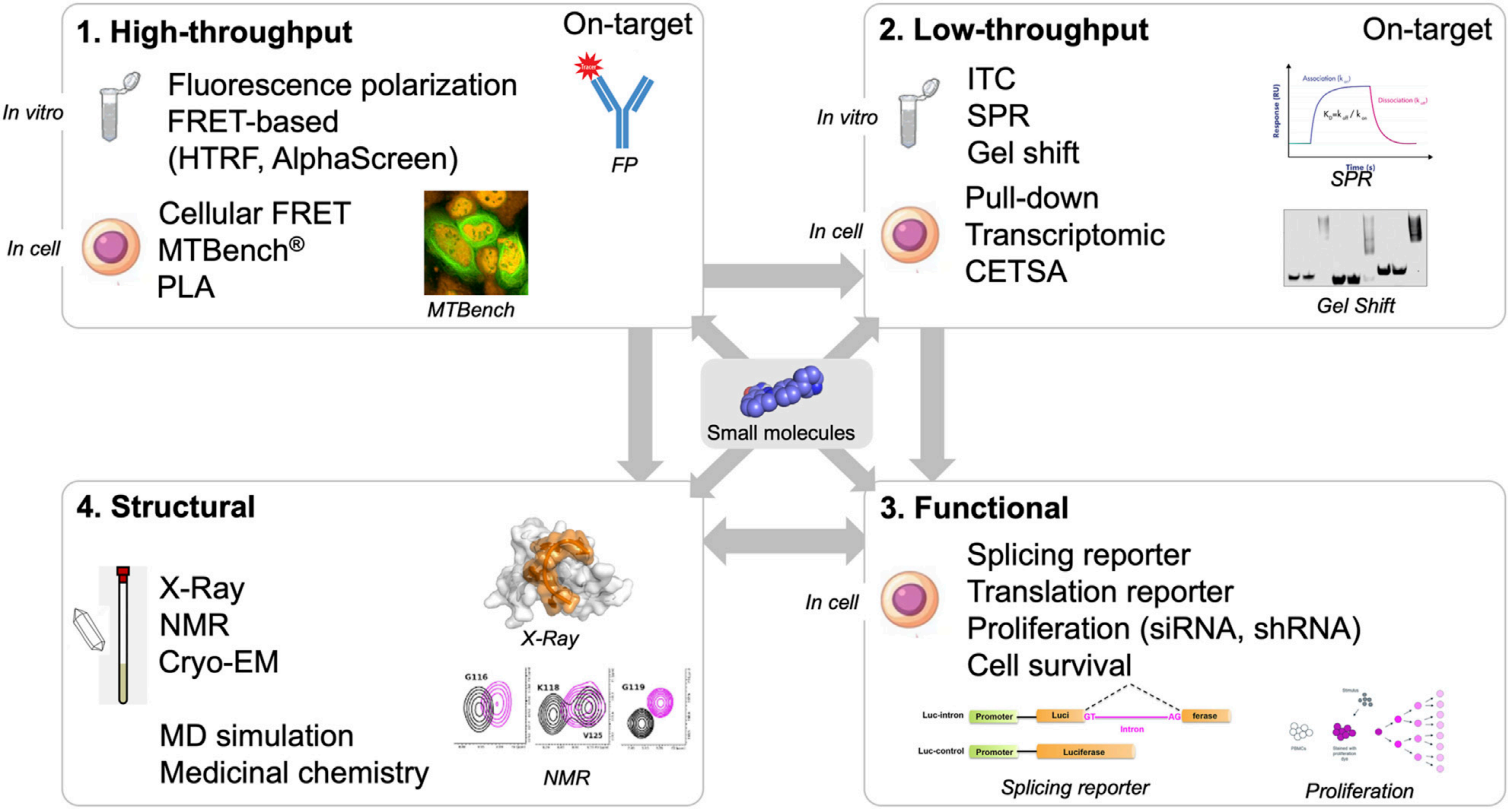
Screening workflow composed by high- and low-throughput screening approaches (*in vitro* and in cell assays), structural and functional validation.

*In silico* approaches could greatly benefit from structural data to identify binding pockets, validate that molecules are on-target *in vitro* (biophysical assays), and provide atomic-level information about the interaction of small molecules with protein and/or RNA. The last point is critical for designing compounds based on derivatives of molecules entering the targeted pocket. With structural data, we can delineate the binding mode of small molecules and identify which amino acid residues and RNA bases are involved in the recognition of the small molecules (Figure 3).

Structural data for proteins interacting with RNA and/or small molecules can be obtained by X-ray crystallography or NMR spectroscopy. X-ray crystallography has been widely used in the drug industry for the development of enzyme inhibitors. However, crystal structures of RBPs interacting with small molecules are scarce (Jagtap et al., 2020). Further research should be undertaken in this direction since X-ray crystallography provides atomic-level information about the binding of small molecules in the targeted pocket. In addition, X-ray crystallography may allow us to gather structural data about ternary complexes in which small molecules would interact with an RNA: protein complex. However, obtaining crystals is not always feasible, especially in the presence of RNA. Furthermore, X-ray structures provide fixed images of inherently dynamic complexes. The most stable interaction with the pocket may not be critical for drug development. Transient, low-energy can also be valuable in drug discovery because they may be easier to target with low-affinity molecules. Targeting intermediary states may have significant consequences for the formation of stable RNA: protein complexes in cells.

Obtaining the structure of RNA: protein complexes from NMR spectra is more tedious than analyzing X-ray data, but it is feasible. However, the main advantage of NMR spectroscopy for drug discovery is its ability to work in liquid to capture dynamic interactions between small molecules, RNA, and protein residues even with low-affinity compounds (El Hage et al., 2023). Many RBPs targeted by small compounds have been analyzed by NMR [e.g., YBX1 cold-shock domain (El Hage et al., 2023), SPF45 UHM domain (Jagtap et al., 2020), U2AF65 (Kobayashi et al., 2022), Lin28 (Wang et al., 2018), HuR (Lal et al., 2017; Manzoni et al., 2018; Wu et al., 2023), Musashi (Lan et al., 2015), TDP-43 (François-Moutal et al., 2019; Nshogoza et al., 2019), IGF2BP1 (Dahlem et al., 2022; Wallis et al., 2022)]. NMR spectroscopy also enables screening many different conditions, such as varying small molecule concentrations and exploring the binding of small molecules with proteins or RNA alone and in complex. Obtaining details about whether small molecules interfere negatively or positively with RNA:protein interactions is also very interesting. For instance, small molecules can serve as chaperones or compete for the binding of a protein to its RNA target.

The use of NMR also presents several challenges. First is the use of proteins with low molecular weight, generally lower than 30–50 kDa, although most RBPs are relatively small. Second is the solubility of RBPs when they harbor self-adhesive and unstructured domains. In this case, self-adhesive domains need to be truncated to perform NMR investigations. The solubility of small molecules can also be an obstacle in NMR experiments. This technique requires relatively high concentrations of products, especially if the affinity of the target is low for compounds. Finally, NMR analysis provides information about binding at the atomic level (chemical shift perturbation and peak disappearance), which cannot be easily translated into binding energy information, apart from defining the binding pocket. Molecular dynamics simulations are an interesting complement to both NMR and X-ray data by delineating possible models of interaction between small molecules, RNA, and proteins that can be probed by mutating key residues that are involved in the putative interactions.

Gathering NMR or X-ray structural data on the interactions between small molecules and RNA:protein complexes is valuable but is not well suited for screening thousands of compounds due to the cost of these methods. Therefore, NMR and X-ray crystallography analyses should be limited to the most promising hits that have been validated *in vitro* by other methods and, if possible, in cells. Besides using structural data to explore the structure-function relationship of small molecules by chemists, NMR and X-ray data serve as substantial evidence that the molecules are on target, even if the occurrence of off-target binding could be significant in cells.

## 4 *In vitro* assays to probe molecules that target RNA: protein interactions

### 4.1 High-throughput *in vitro* assays

High-throughput assays are critical components of the screening pipeline (Figure 3). *In silico* approaches can rarely reduce the number of putative hits down to thousands of compounds. The number of compounds selected *in silico* can be dramatically reduced if the selection is based on general criteria such as solubility, size, toxicity, and membrane crossing. Even if structural information based on NMR or X-ray crystallography data are used, the number of compounds selected is still high, from hundreds to thousands at least. With high-throughput assays, thousands of compounds can be screened. One general problem with the use of high-throughput assays *in vitro* is the solubility of RBPs. As in the case of using NMR and X-ray crystallography, the self-adhesive domains of the RBPs have to be removed.

The two most widely used *in vitro* high-throughput assays for RNA:protein interactions are described below and make use of recombinant proteins combined to short RNA molecules.

#### 4.1.1 Fluorescence polarization assay

Fluorescence polarization requires the use of purified protein, RNA, or RNA: protein complex. When light interacts with a RNA:protein complex, changes in polarization can be recorded in assay plates such as 384-well plates (Smith and Eremin, 2008). When small molecules interact with the RNA:protein complexes, tiny changes in fluorescence polarization can be recorded and scored. Due to its simplicity of use and sensitivity fluorescence polarization is one of the most commonly used methods to identify hit compounds from primary screens. It has already been used for probing small molecules targeting let-7: Lin28 (Borgelt et al., 2021), UHM domain (Jagtap et al., 2020), HuR (Wu et al., 2015; Lal et al., 2017), and others. It should be noted that the intrinsic fluorescence of some small compounds can limit the use of this approach. In addition, labeling protein/RNA with a fluorescent group or using a competitive fluorescent probe have to be used to detect changes in fluorescence polarization.

#### 4.1.2 FRET-based assays

The short range energy transfer between a pair of fluorescence labels (probes) is inversely proportional to the sixth power of the distance between two dye molecules (Roos et al., 2016). Thus, FRET changes the fluorescence signal to detect with high sensitivity whether the separation distance between two biomolecules is shorter than few nanometers. To detect RNA:protein interactions, FRET assays use a protein and its RNA target, each tagged with a different fluorescent probe displaying recovery in their emission spectra of donor and acceptor. In turn, RNA must also be labelled with fluorescent or quencher molecules. Since RBPs bind to long RNAs in many different places, at least *in vitro* without competitors, long RNAs with which many RBPs can be associated are not a good choice for FRET-based assays. In this case, many RBPs in the complex would be located some distance away from the RNA fluorescence label, which is generally located at the 3′ or 5′ ends of the RNA. Therefore, FRET assays require labeled proteins combined with short labeled RNA. HTRF assays and AlphaScreen are high-throughput alternatives to screen inhibitors of RNA:protein interaction (Hoskins et al., 2014; Pedram Fatemi et al., 2015; Wu, 2020) that also use short-range fluorescence transfer. For instance, AlphaScreen was used to screen molecules targeting RNA:HuR interaction in 384-well plates (D’Agostino et al., 2013).

### 4.2 Low-throughput *in vitro* assays

Complementing high-throughput assays, relatively low-throughput assays can be used to confirm the efficacy of selected hits *in vitro* and to provide additional information regarding the interference of small compounds with RNA:protein interactions or the binding of selected compounds to protein and/or RNA.

#### 4.2.1 Isothermal titration calorimetry

Isothermal titration calorimetry (ITC) is a sensitive method to explore the thermodynamic properties of proteins or RNAs interacting with small molecules, as well as RNA:protein interactions (Feig, 2009). From a single ITC experiment, one can obtain binding and thermodynamic parameters such as association constant (K_A_), stoichiometry (n), enthalpy (ΔH), and entropy (ΔS) changes. While this method is very useful, the sensitivity for the analysis of low affinity compounds (>10 µM) is limited. ITC is also not suitable for screening many compounds because it requires a large amount of proteins and RNAs, which also increases the cost of the screen. In addition, ITC has been rather used for investigating two-component interactions such as RNA: small molecules or RNA: protein interactions. To screen small molecules interacting with protein: RNA complex, the reading can be more complicated since a decrease/increase of RNA: protein interactions would provide additional changes in the thermodynamic parameters.

#### 4.2.2 Surface plasmon resonance

To probe the interactions of proteins, RNA, and small molecules, surface plasmon resonance (SPR) is a sensitive technique that uses biomolecules immobilized on a solid support. SPR assays are particularly useful for studying RNA ligands (Vo et al., 2019) and RNA:protein interactions (Katsamba et al., 2002) and measures affinities and kinetics in real time. SPR is also a label-free technique. The major drawback to this approach is the immobilization of the proteins or RNAs on solid supports, which may affect the interaction of small molecules with the RNA:protein complex. In addition, like ITC, SPR, has been widely used for investigating two-component interactions but detecting whether small molecules interact with RNA:protein complex is most probably challenging.

#### 4.2.3 Gel shift assays

Gel shift assays, in which RNA:protein complexes are run in acrylamide or agarose gels, have a long history of use by biochemists and biologists. Small molecules can change the electrophoretic mobility of the targeted RNA:protein complex (Yakhnin et al., 2012). However, this method is suitable for compounds with high affinity (<1 µM) that could interfere with the binding of the protein to RNA, a scenario that is rarely the case for inhibitors of RNA:protein interactions.

## 5 Cellular assays

### 5.1 High-throughput cellular assays to probe molecules that target RNA: protein interactions

**FIGURE 4:**
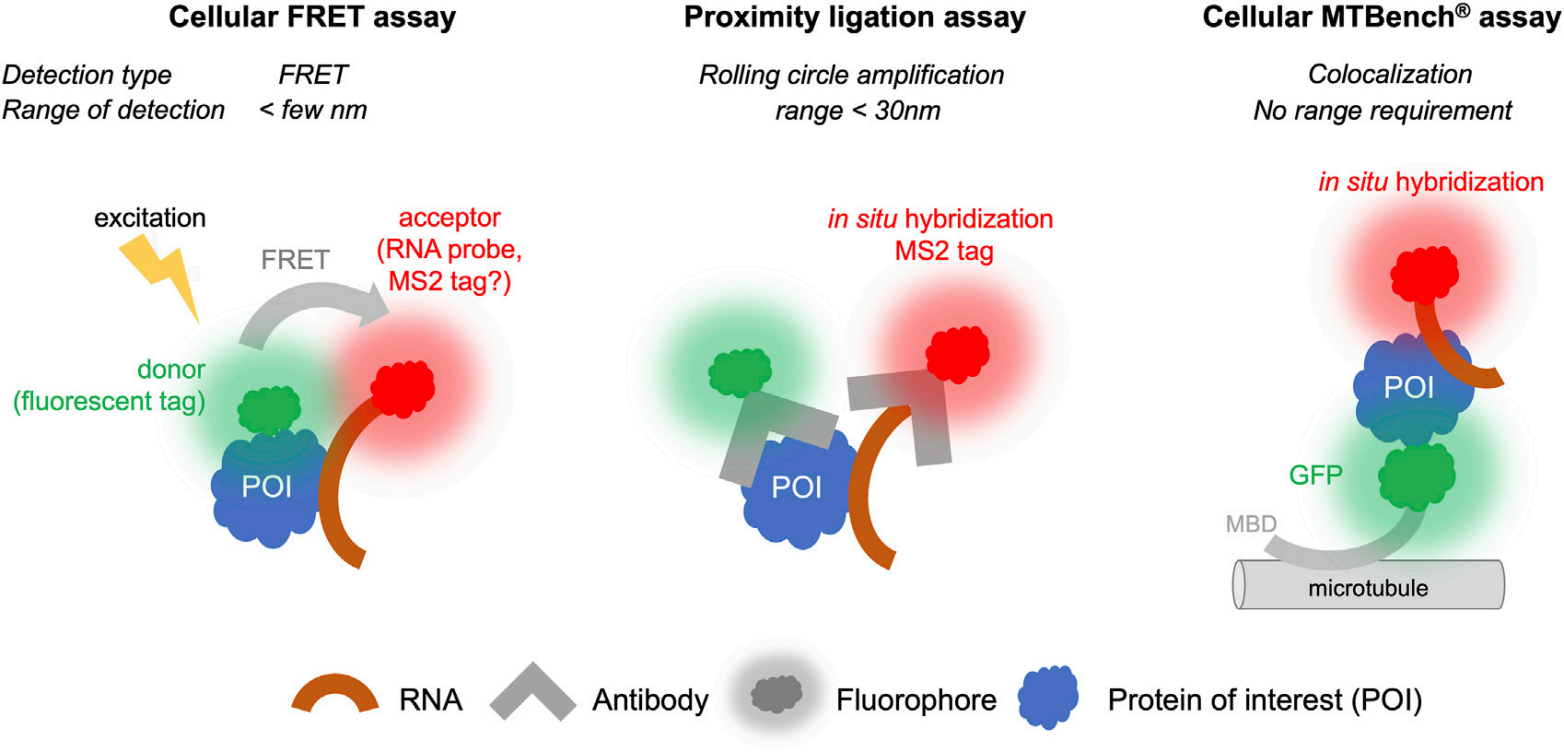
Proximity Ligation, FRET, and MTBench^®^ assays able to screen chemical library targeting RNA: protein interactions in cells.

Although *in vitro* experiments are useful, ensuring that chemical compounds targeting RNA:protein interactions are indeed on target in cells is essential. Small molecules that have been selected *in vitro* from large libraries rarely have high affinity for RNA, RBPs, or RNA:protein complexes. Generally, compound affinities are several micromolar or even hundreds of micromolar. In the cell, these compounds may not cross cell membranes and may be degraded in the cell or in the extracellular environment. In addition, small molecules can also nonspecifically interact with other biomolecules to divert them from the intended RNA: protein complex. Finally, *in vitro*-selected molecules may not bind to their RNA:protein target in cells due to different folding of the RNA: protein complex in a cellular context that cannot be reproduced *in vitro*. Therefore, an interesting strategy would be to screen directly in cells whether selected compounds bind to the target in cells. However, only a very limited number of methods can provide this information (Figure 4).

#### 5.1.1 Cellular FRET assays

FRET is technically amenable in cells to provide high-throughput data, although there are a limited number of examples in which FRET has been used in cells for screening. However, while the protein target can be labeled with a fluorescent probe, it is more difficult to label an RNA molecule in the cell than a protein. For this reason, FRET is more suitable for detecting protein: protein interactions than RNA: protein interactions (Day and Davidson, 2012), although some developments have been proposed to detect RNA: protein interactions (Huranová et al., 2009; Alam et al., 2023). RNAs exogenously expressed in cells can be detected by adding stem-loops to their 3′ends, which will be recognized by a fluorescent protein such as the MS2 protein (Tutucci et al., 2018). It is also possible to detect endogenous RNA by *in situ* hybridization with fluorescent probes to perform FRET experiments, though the copy number of mRNA molecules per cells is quite low (<100) for most genes. The further development of FRET for RNA:protein interactions to date has been limited by the constraint that fluorescent molecules used as labels for proteins and RNAs must be located in close proximity, i.e., less than a few nanometers. RBPs can bind to many different RNAs in cells and often at different positions on the same RNA. For example, transcriptomic analyses (Van Nostrand et al., 2020) have indicated that most mRNA-binding proteins can bind to dozens of sites on the same mRNA. If *in situ* hybridization is used to target a specific site on this RNA, the FRET signal will only be recorded when the associated RBP is close to the fluorescent label of the probe used for *in situ* hybridization. Furthermore, the attachment of the RBP to other sites of the same RNA may disturb the analysis and the sensitivity of detection. Nevertheless, FRET is an established and highly sensitive technique. Future developments can be envisaged to circumvent these obstacles and allow the use of this technique for applications involving RNA:protein interactions (Figure 4) (Huranová et al., 2009).

#### 5.1.2 Proximity ligation assays

Proximity ligation assays (PLA) use antibodies that recognize endogenous proteins to detect their spatial proximity based on an amplification process. The amplification relies on a hybridization step followed by a DNA amplification step to incorporate fluorescent probes. When the separation distance between the two proteins is lower than ∼30 nm, the PLA signal is detected via the visualization of fluorescent spots in cells by microscopy. This method, like FRET, is especially suitable for detecting protein: protein interactions. It can be adapted to detect RNA: protein interactions if RNA can be detected with a dye, for example, by using an RNA probe labeled with digoxigenin to enable the detection of endogenous RNAs using antibodies. However, to date, very few studies have benefited from this method to explore RNA: protein interactions (Roussis et al., 2016; Zhang et al., 2016; George et al., 2022). First, the cost of secondary antibodies and reagents to achieve amplification is expensive, and it is therefore unlikely that high-throughput screens could be used under these conditions. Second, when the RNA target is present in low number of copies, the interaction will be certain RNAs, such as non-coding RNAs RNAs and mRNAs, are present with a relatively limited number of copies per cell (a few tens of copies for most mRNAs). Given the low number of mRNA copies, the interaction will be detected with a low occurrence that can decrease the sensitivity of the technique and pose challenges for obtaining quantitative data. Nonetheless, the possibility of detecting interactions with endogenous proteins and endogenous RNAs remains a significant advantage and suggests that this approach has potential for further advancements (Figure 4).

#### 5.1.3 MTBench assay

MTBench is a technique that uses microtubules as an intracellular platform to detect protein:protein (Boca et al., 2015; Rengifo-Gonzalez et al., 2021) and RNA:protein interactions (El Hage et al., 2023) in the cell. A protein is introduced onto microtubules in the cell by fusing it to a microtubule-binding domain and a fluorescent tag for detection. The bait protein then attracts RNAs onto the surface of the microtubules, which are detected using *in situ* hybridization. The greater the number of RBPs on the microtubules, the greater the number of RNAs are detected. The enrichment of RNA on the microtubules is then recorded as a function of the number of bait proteins, providing an interaction score and a direct estimate of the protein’s affinity for RNA in cells. This method is relatively simple to set up and has already been validated in 96-well plates for detecting interactions with endogenous mRNAs (El Hage et al., 2023). Adapting this method for plates with a larger number of wells is theoretically possible. However, this method requires fusion of protein with a microtubule-binding domain, which may affect the binding of protein to RNA in some cases. Controls must performed to address this possibility, as demonstrated with YBX1, FUS, and HuR proteins (El Hage et al., 2023). Additionally, the interaction is measured in the cytoplasm, which may not reflect nuclear RBP interactions. Furthermore, only poly (dT) probes have been used for detection of endogenous mRNAs to date. Whether the sensitivity of this method is sufficient to detect interactions between a protein and a specific RNA, which often has a limited copy number, remains the subject of ongoing research. Nevertheless, a screen has been conducted to identify compounds affecting the interaction between YBX1 protein and mRNA in 96-well plates (El Hage et al., 2023). Given its simplicity and sensitivity, it is likely that this method can be further developed for high-throughput screening of specific RNA:protein complexes (Figure 4).

### 5.2 Low-throughput cellular assays

#### 5.2.1 Cellular thermal shift assay

In the cellular thermal shift assay (CETSA), cell lysates or cell suspensions are heated at different temperatures. The stability of RBPs in the soluble fraction is then assessed by western blot analysis or by mass spectrometry if the target protein of the molecule is unknown. Molecules targeting RBPs may alter the stability of RNA: protein complexes (Dahlem et al., 2022; Malaney et al., 2022; Zhou et al., 2023). This method is simple and easy to use. However, because cells or cell extracts are heated, the interaction between the protein target and RNA can be affected. Since this method does not directly probe whether small molecules affect RNA: protein interactions, it cannot exclude off-target effects of the molecules that could increase or decrease the thermal stability of the protein or stabilize/destabilize its interaction with RNA in cells. Despite these considerations, CETSA is a valuable assay for providing complementary information about whether selected compounds are on target.

#### 5.2.2 Pull-down assay

Pull-down assays from cells treated with biotinylated molecules or biotinylated control molecules can be analyzed by western blot assays or by mass spectrometry (Tailor et al., 2021; D’Agostino et al., 2015). When the affinity between the molecules and the target protein is strong, an interaction can be detected by identifying the protein in the pull-down fraction. Weak interactions are less likely to be detected. In addition, the biotinylation of small molecules required for pull-down assays may significantly alter their activity, leading to biased results that may not accurately reflect the target of the non-biotinylated molecules. Finally, functionalizing small molecules for pull-down assays is a challenging task. Whether a compound interferes with the binding of the protein to RNA cannot be demonstrated by this method. In most applications, pull-down assays are used to identify proteins that could be targeted by known compounds that have shown promising properties for therapeutic use. Alternatively, RNA or RNA-binding protein can be pulled down to probe whether small molecules can interfere with RNA:protein interactions. However, redistributions of RNA and RNA-binding proteins can occur after cell lysis. In addition, small molecules also have to be kept during the pull-down procedures unless the affinity of the small molecules for the target is very high.

#### 5.2.3 Transcriptomic analysis

Several methods have been developed to detect intracellular interactions between RBPs with RNA at a genomic scale (Tome et al., 2014). The most frequently used method, CLIP (Kishore et al., 2011), identifies the protein attachment sites on genomic RNA by cross-linking RNA:protein complexes, followed by treatment with an RNAse to retain only the regions of the RNA protected by the protein of interest. These methods are highly precise and can reveal whether molecules alter the interactions between an RBP with genomic RNA. This approach can also define whether small molecules affect protein binding to specific RNAs and to particular sequences or secondary structures. However, using such a method can be laborious and expensive (Garzia et al., 2017). Thus, transcriptomic analyses are recommended more specifically for assessing a limited number of hits that have been validated by other methods.

### 5.3 Functional cellular assays

Functional assays are also critical to further investigating the efficacy of small molecules. These assays can measure whether translation, splicing, transcription, or other cellular functions controlled by specific RNA: protein interactions can be modified by small molecules.

Functional assays may involve the use of specific gene reporters where the RBP or RNA targets play a direct role. For example, to probe the inhibition of the complex formed by let-7 microRNA and Lin28 protein, dual luciferase reporter assays were used with eight tandem let-7 recognition sites in the 3′UTR to confer sensitivity to let-7 microRNA levels (Wang et al., 2018). Splicing reporters specific to a particular protein (e.g., TDP-43, which controls the skipping of CFTR exon 9), can also be used (Lukavsky et al., 2013). Actinomycin chase experiments can also be used to test whether a compound interfered with the binding of target protein to mRNA which may results in a varying half-life for mRNA targets. However, in most cases, more general features such as cell proliferation, stress resistance, or cellular phenotypes such as neurite outgrowth are used to assess the impact of small molecules. In functional assays, the expression of the targeted RBP can be decreased by using shRNA, siRNA, or CRISPR-Cas9 technology, and/or expression of the RBP (or a mutant with a disrupted binding pocket) can be added back. If the molecules fail to exhibit any activity when the targeted RBP expression is increased or after a mutation is introduced in the binding pocket, the results may be considered conclusive. However, even if functional assays are necessary for evaluating the consequences of disrupting RNA: protein interactions on cellular functions, the results come with significant uncertainty regarding whether the molecules are indeed on-target. RBPs are associated with multiple functions, complicating the task of correlating small molecule effects with the functions of the target (Lukavsky et al., 2013). In addition, when a RBP expression is silenced, cells can compensate for the missing protein by altering the expression level of many different RBPs. It is also important to note that small molecules targeting RNA:protein interactions generally have weak affinities, at least during the initial hit identification phase prior to medicinal chemistry modifications. Consequently, a high concentration of the compound is often necessary to observe an effect on protein, RNA or RNA: protein complex functions in cells. Under such conditions, toxicity and off-target effects become additional parameters to consider, making the interpretation of functional assays results more challenging and highlighting the importance of confirming whether small molecules are on target in cells.

## 6 Perspectives

### 6.1 Targeting RNA directly with small molecules

RNA is now recognized as a major target that could pave the way for new therapies in various human diseases (Table 1), including cancers (Kechavarzi and Janga, 2014; Sebestyén et al., 2016) and certain neurodegenerative diseases (Renoux and Todd, 2012; Harrison and Shorter, 2017; Nedelsky and Taylor, 2019; Gebauer et al., 2021; Hill and Meisler, 2021). Initial successes have already been achieved by targeting RNAs with ASOs. Validated targets can modulate mRNA splicing (Palacino et al., 2015; Ratni et al., 2018) or modify the expression of factors implicated in human disease (Havens et al., 2013; Havens and Hastings, 2016; Montes et al., 2019; Hill and Meisler, 2021), such as inducing SMN2 expression in spinal muscular atrophy (Hua et al., 2008; Hua et al., 2010). These promising results serve as proof of concept, demonstrating that specific RNA targeting can benefit patients. However, due to cost constraints and challenges related to administration methods, the use of ASOs is currently limited to a relatively small number of patients. Therefore, small molecules may be a credible alternative to usher in a new class of molecules targeting RNAs. Many research groups have shifted their focus toward developing compounds that directly target RNA. This strategy holds great promise, especially when targeting structures involved in human diseases, such as GC repeats (Meyer et al., 2020), triplet repeats (Grant et al., 2006; Khan et al., 2019), or G-quadruplexes (Simone et al., 2018) in neurodegenerative diseases. In such cases, RNA adopts unique structures or contains specific sequences that offer a basis for specificity for small molecules with therapeutic potential. However, in most cases, the sequences targeted by these small molecules encompass only a few nucleotides, which are highly repetitive in the human genome. This redundancy complicates the development of specific molecules. Nevertheless, targeting RNA presents an intriguing challenge that must be addressed given its significant therapeutic potential for numerous human diseases.

**TABLE 1:** Examples of FDA approved and clinical trial of small molecules that bind to RNAs with applications in oncology and neurology (Childs-Disney et al., 2022).

Therapeutic area	Associated disease	Compound/institution	RNA target	Mode of action	Stage of development	Ref
Oncology	Triple-negative breast cancer and chronic myelogenous leukaemia	Zotatifin (eFT226)/eFFECTOR Therapeutics & Inception Therapeutics	Polypurine sequences in the 5′UTR of a subset of oncogenic mRNAs	Inhibits translation initiation by clamping eIF4A to polypurine RNA sequence in the 5′UTR	Phase I–II clinical trial: NCT04092673	Ernst et al. (2020)
Neurology	SMA	Risdiplam/Roche and PTC Therapeutics	SMN2 pre-mRNA exon 7–intron junction	Promotes exon inclusion by stabilizing the binding of the splicing machinery	FDA approved	Ratni et al. (2018)
Neurology	SMA	Branaplam	SMN2 pre-mRNA exon 7–intron junction	Promotes exon inclusion by stabilizing the binding of the splicing machinery	Phase I/II clinical trial (for SMA: NCT02268552; for HD: NCT05111249)	Palacino et al. (2015)

### 6.2 Targeting only RBPs in certain human diseases

Some RBPs are considered as target because of their involvement in human diseases including cancer (Cen et al., 2023) and neurodegeneration (Conlon and Manley, 2017). For small molecules directly targeting RBPs, and thus indirectly affecting RNA, additional challenges arise. Although numerous RBPs have been selected as targets for molecule in drug discovery, such as eIFAa, an RNA helicase involved in cancer (Ernst et al., 2020), few small molecules exhibit high selectivity. Most small molecules have moderate effects and affinities greater than 1 µM (Minuesa et al., 2019; Wu, 2020; Tan et al., 2022). Unlike domains with enzymatic activity, most RBPs have no well-defined hydrophobic pocket. Most RNA-protein interactions rely on electrostatic interactions involving structured domains as well as low-complexity domains [e.g., repetitive arginine and glycine/serine residues (RGG and SR domains)]. Aromatic residues, which interact directly with RNA bases, contribute some specificity to certain RNA sequences. Such interactions primarily occur within structured domains (e.g., RRM, KH, ZnF) and do not require the presence of hydrophobic pockets. Consequently, RBPs have often been considered poorly druggable (Wu, 2020). Therefore, while recent progress has been encouraging, targeting RBPs remains a formidable task. Another complication arises from multifunctional nature of RBPs. Genome-wide studies conducted at the transcriptomic level have revealed that RBPs bind to numerous RNAs in cells (Burd and Dreyfuss, 1994). Directly targeting RBPs may therefore lead to unintended side effects that limit the therapeutic application of the developed molecules. However, certain RBPs are clear targets in their own right, such as TDP-43 and FUS, for which pathological mutations have been identified in specific neurodegenerative diseases. These RBPs also form cytoplasmic inclusions in neurons in patients (Lagier-Tourenne et al., 2012). It is therefore essential to anticipate the risks associated with targeting an RBP before selecting it as a target. Tests should be conducted in cellular contexts as well as in animal models to evaluate the consequences of globally altering the functions of an RBP using CRISPR, ASOs, siRNA, or shRNA.

### 6.3 Targeting RNA: protein interfaces to enhance specificity and to correct/modify a specific function displayed by the chosen RNA: protein complex

Targeting RNA: protein interfaces offers several advantages. First, protein targets provide diversity in amino acid sequences and structures, both of which are limited in RNA molecules. RNA, when complexed with proteins, can reveal druggable pockets that are not as abundant in RBPs in isolation. Another advantage is the ability to target specific functions associated with RNA: protein interactions. One well-studied example is the interaction between let-7 and Lin28, which controls cell differentiation (Heo et al., 2008). Lin28 binds to a large number of RNAs, including cytoplasmic mRNAs and nuclear RNAs in nucleoli (Wilbert et al., 2012). Consequently, it may be interesting to specifically target the let-7: Lin28 complex while avoiding interference with the mRNA-related functions of Lin28. Splicing factors also bind to specific sequences on introns or exons. Developing molecules that alter the splicing of specific mRNAs represents an interesting strategy for splicing corrections using small molecules. Transcriptomic data analysis has made it possible to identify and analyze binding sites of RBPs on pre-mRNAs (Sanford et al., 2009), facilitating investigations into whether RBP binding at specific pre-mRNA binding sites can modulate splicing outcomes as expected. A similar strategy can be employed to correct translation by targeting specific mRNAs to control the expression of encoded proteins. Additionally, microRNAs or certain long non-coding RNAs can be targeted by using RBPs that bind to non-coding RNAs to regulate their biogenesis, stability, or functions. Notably, targeting long non-coding RNAs in many cancers has shown promise (Bhan et al., 2017), although it currently lacks identified small molecules to drive further research. Ongoing research may provide interesting results such as the direct targeting of G4C2 hairpins involved in neurodegenerative diseases (Wang et al., 2019). In this context, using RNA: protein interfaces as targets may provide an interesting and promising strategy to target non-coding RNA in human diseases.

While the prospect of targeting RNA: protein interactions holds considerable appeal, many challenges remain to be addressed. Identifying pockets in which molecules can interfere with RNA: protein interactions is a crucial first step. If some compounds have already been identified, extensive screens based on structural data and *in silico* selection of molecules must be conducted to expand the repertoire of molecules that can target RNA: protein interfaces. *In vitro*, there is currently limited information about the selectivity of proposed molecules with respect to RNA. Studies should encompass different RNA sequences and secondary structures to assess whether molecules exhibit distinct effect in different contexts. These aspects must be addressed *in vitro* and *in silico* through molecular dynamic simulations to determine whether medical chemistry modifications can increase the specificity towards specific RNA targets. Finally, beyond functional cellular data indicating whether molecules alter RNA functions, it is essential to confirm whether molecules indeed target the intended RNA: protein interactions in a cellular context. Few methods allow for the measurement of RBP with RNAs in cells, and even fewer can detect interactions with specific RNAs. Ideally, the detection of these specific RNA: protein interactions should be amenable to high-throughput screening involving thousands of selected compounds or assessing specificity towards different RNAs.
