# Supplementary material for: Using the structural diversity of RNA: protein interfaces to selectively target RNA with small molecules in cells: methods and perspectives
Source: Front Mol Biosci. 2023 Nov 16;10:1298441. doi: 10.3389/fmolb.2023.1298441 (PMC10687564; doi:10.3389/fmolb.2023.1298441)
Supplement: Supplementary file 1 [file Presentation1.PPTX]

## Slide 1
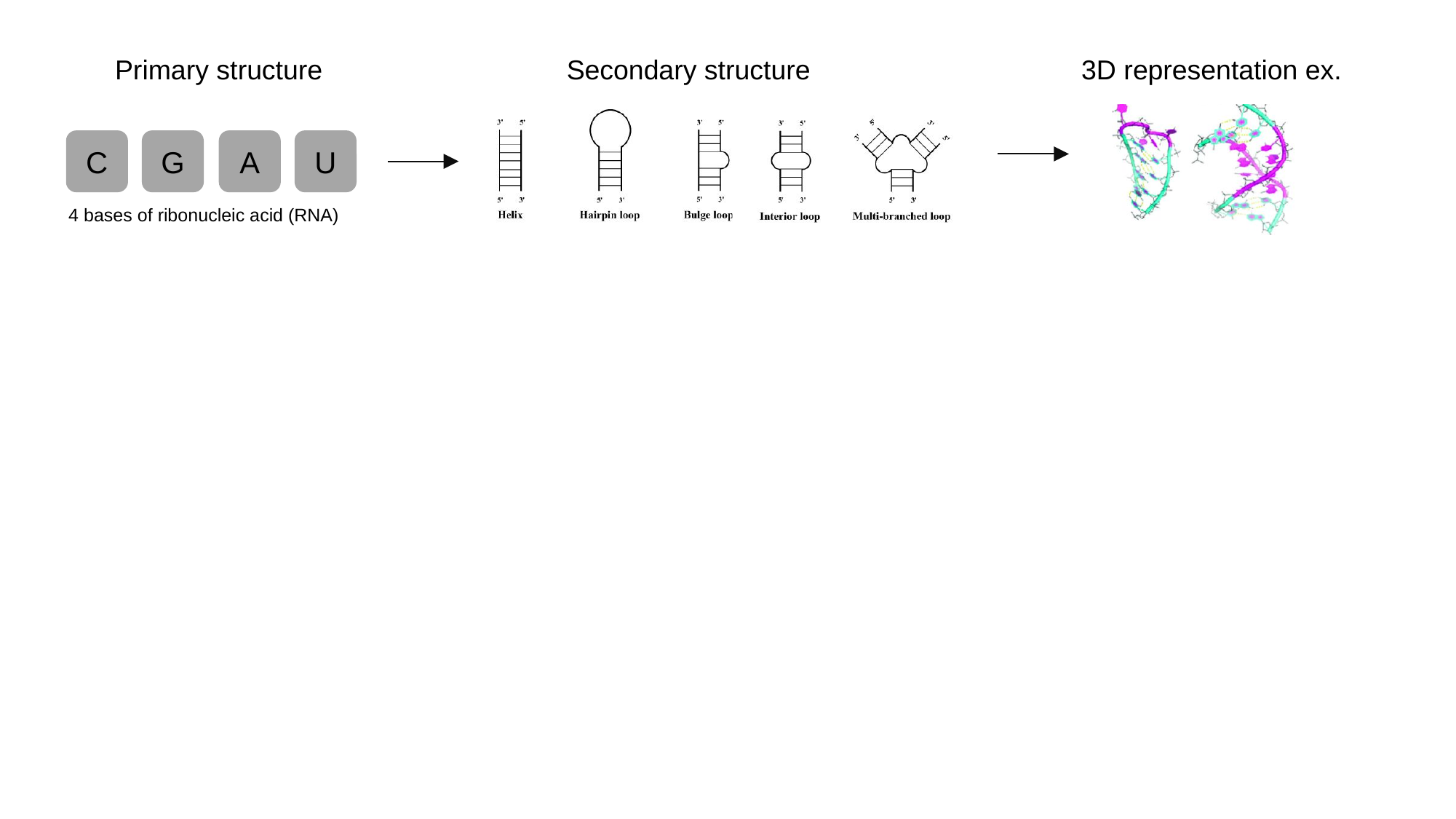

Figure 1.
Primary structure
Secondary structure
3D representation ex.
A
C
G
A
U
4 bases of ribonucleic acid (RNA)
B
C
D

## Slide 2
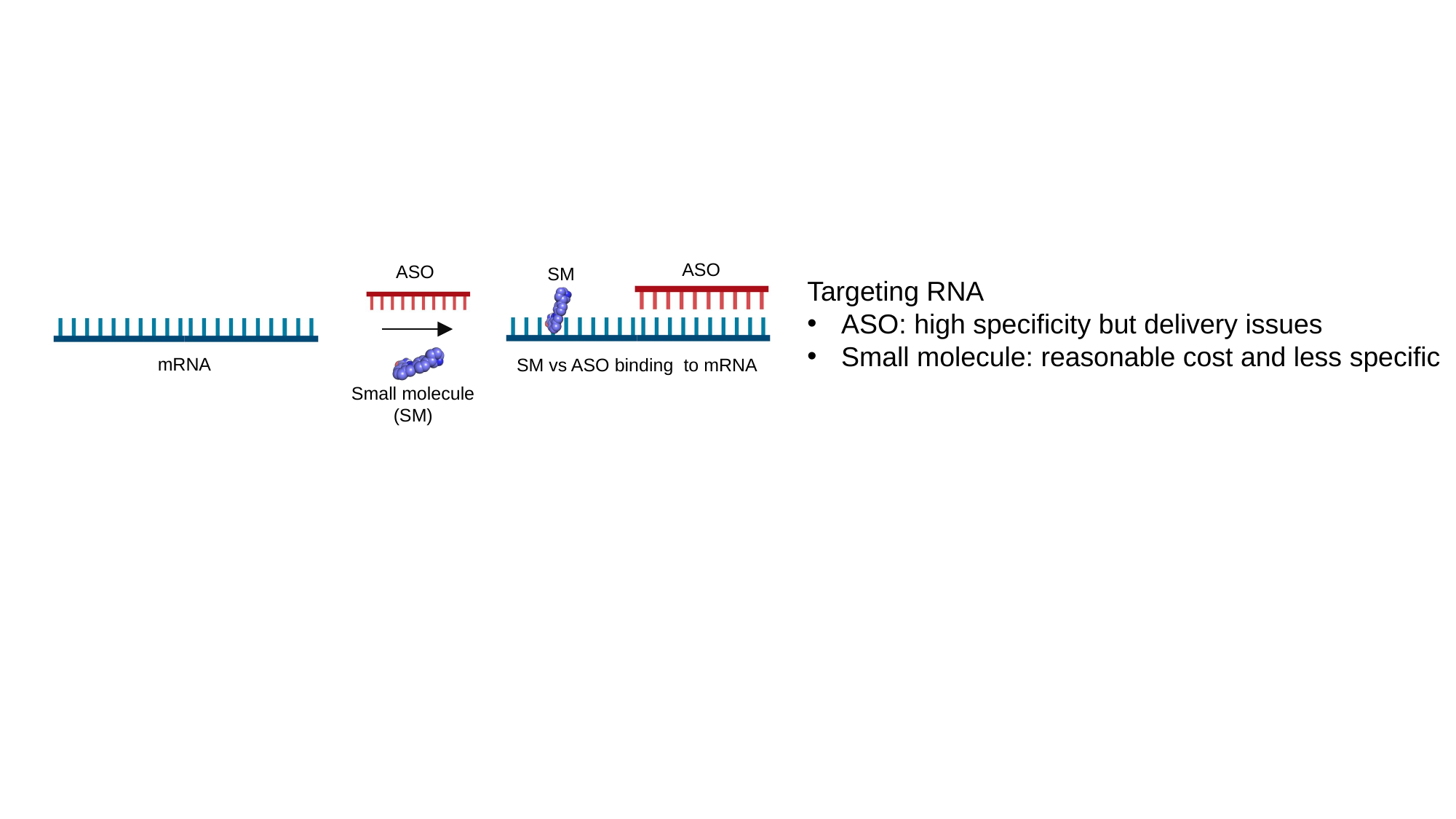

Figure 1.
A
ASO
B
ASO
SM
Targeting RNA
ASO: high specificity but delivery issues
Small molecule: reasonable cost and less specific
mRNA
SM vs ASO binding to mRNA
Small molecule
(SM)
C
D

## Slide 3
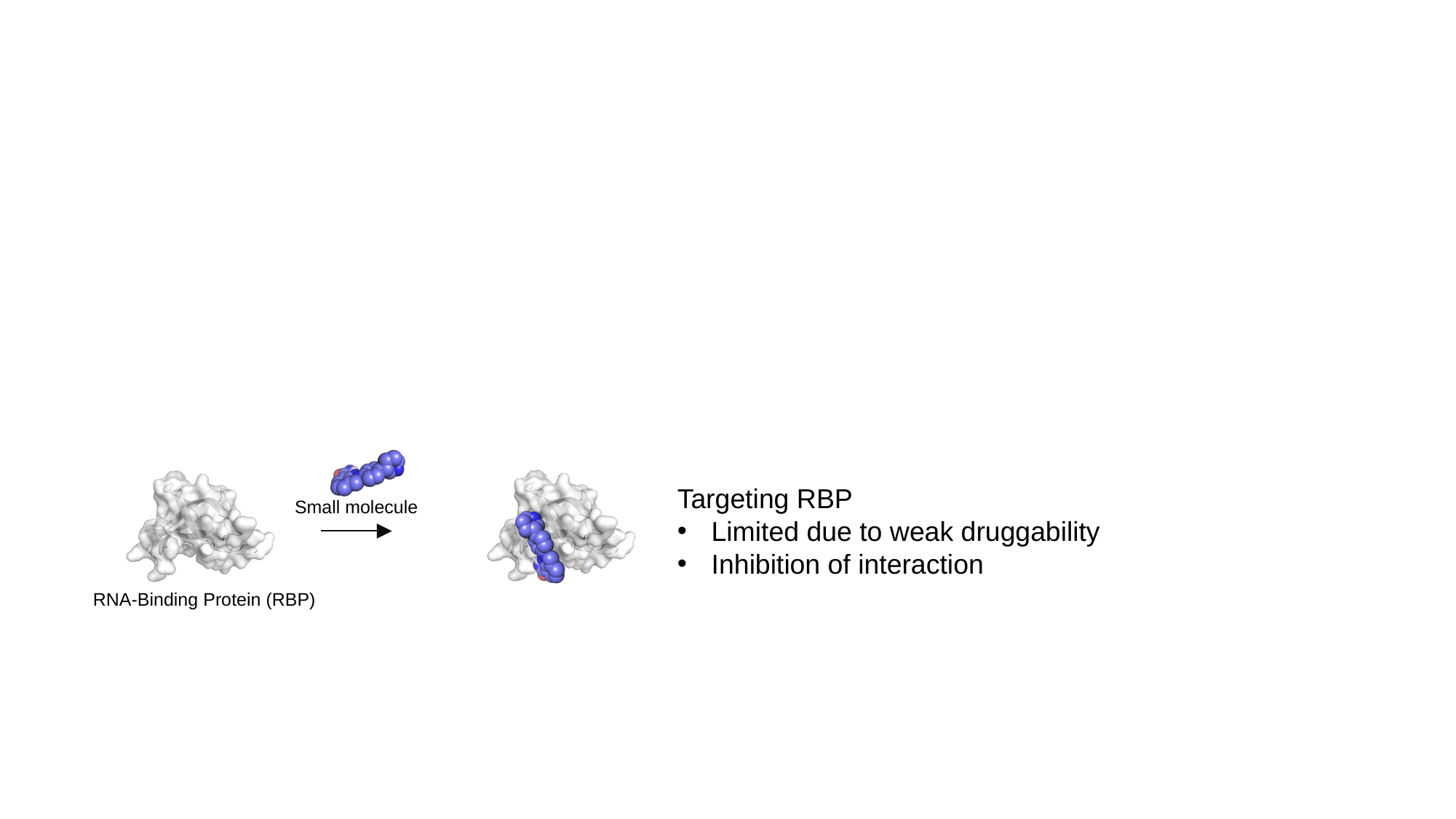

Figure 1.
A
B
C
Targeting RBP
Limited due to weak druggability
Inhibition of interaction
Small molecule
RNA-Binding Protein (RBP)
D

## Slide 4
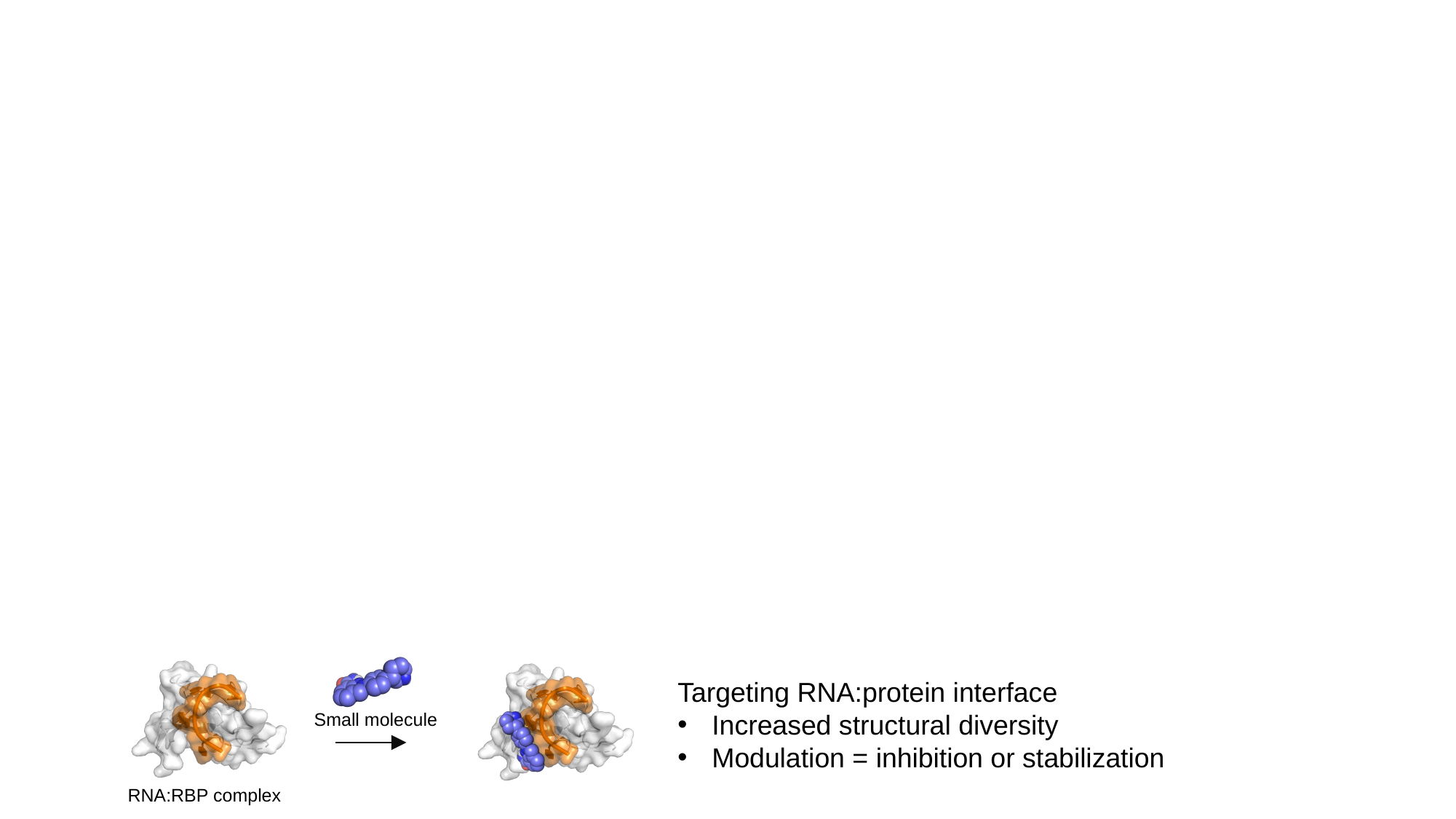

Figure 1.
A
B
C
D
Targeting RNA:protein interface
Increased structural diversity
Modulation = inhibition or stabilization
Small molecule
RNA:RBP complex

## Slide 5
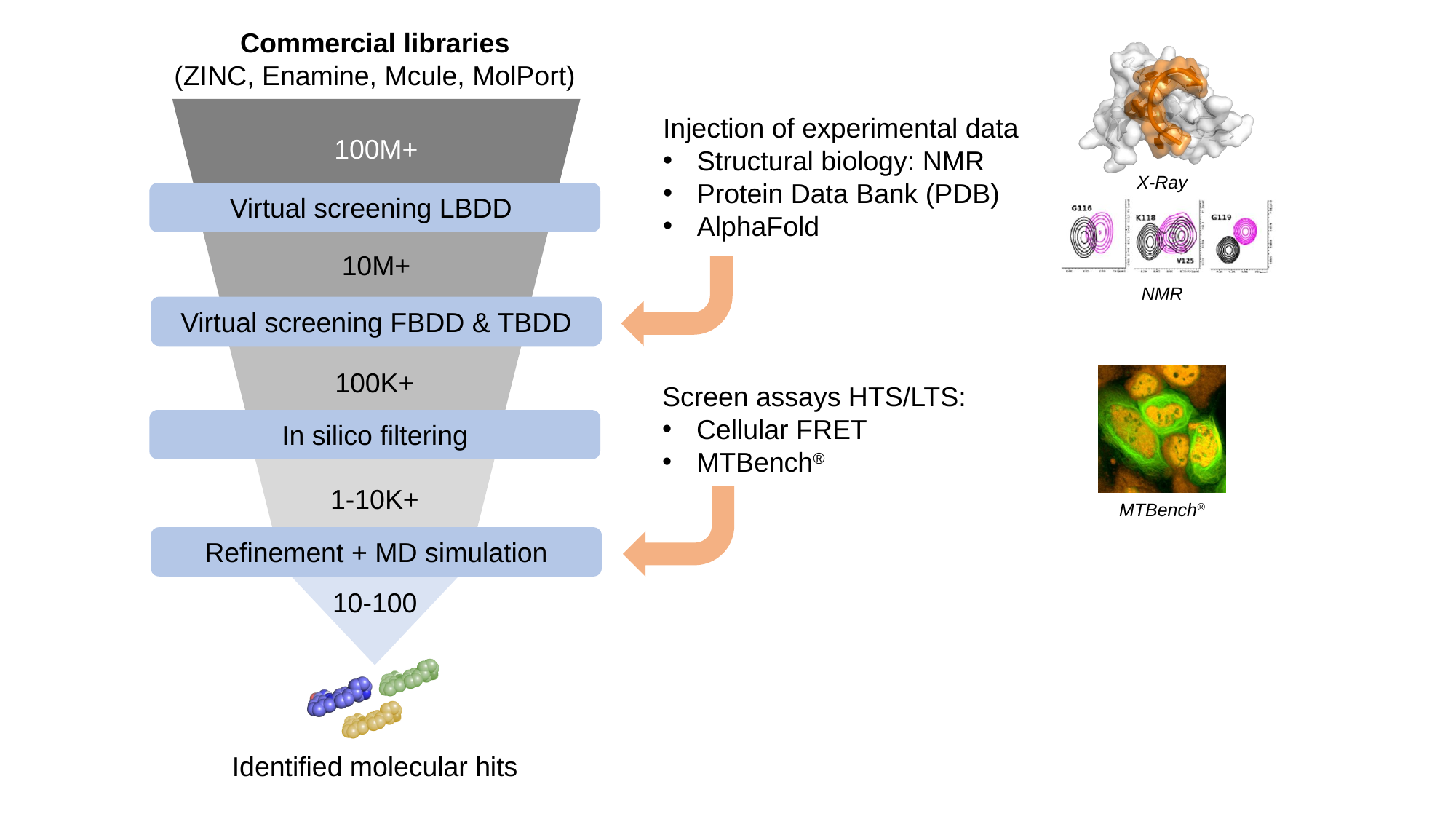

Figure 2.
Commercial libraries
(ZINC, Enamine, Mcule, MolPort)
Injection of experimental data
Structural biology: NMR
Protein Data Bank (PDB)
AlphaFold
100M+
X-Ray
Virtual screening LBDD
10M+
NMR
Virtual screening FBDD & TBDD
100K+
Screen assays HTS/LTS:
Cellular FRET
MTBench®
In silico filtering
1-10K+
MTBench®
Refinement + MD simulation
10-100
Identified molecular hits

## Slide 6
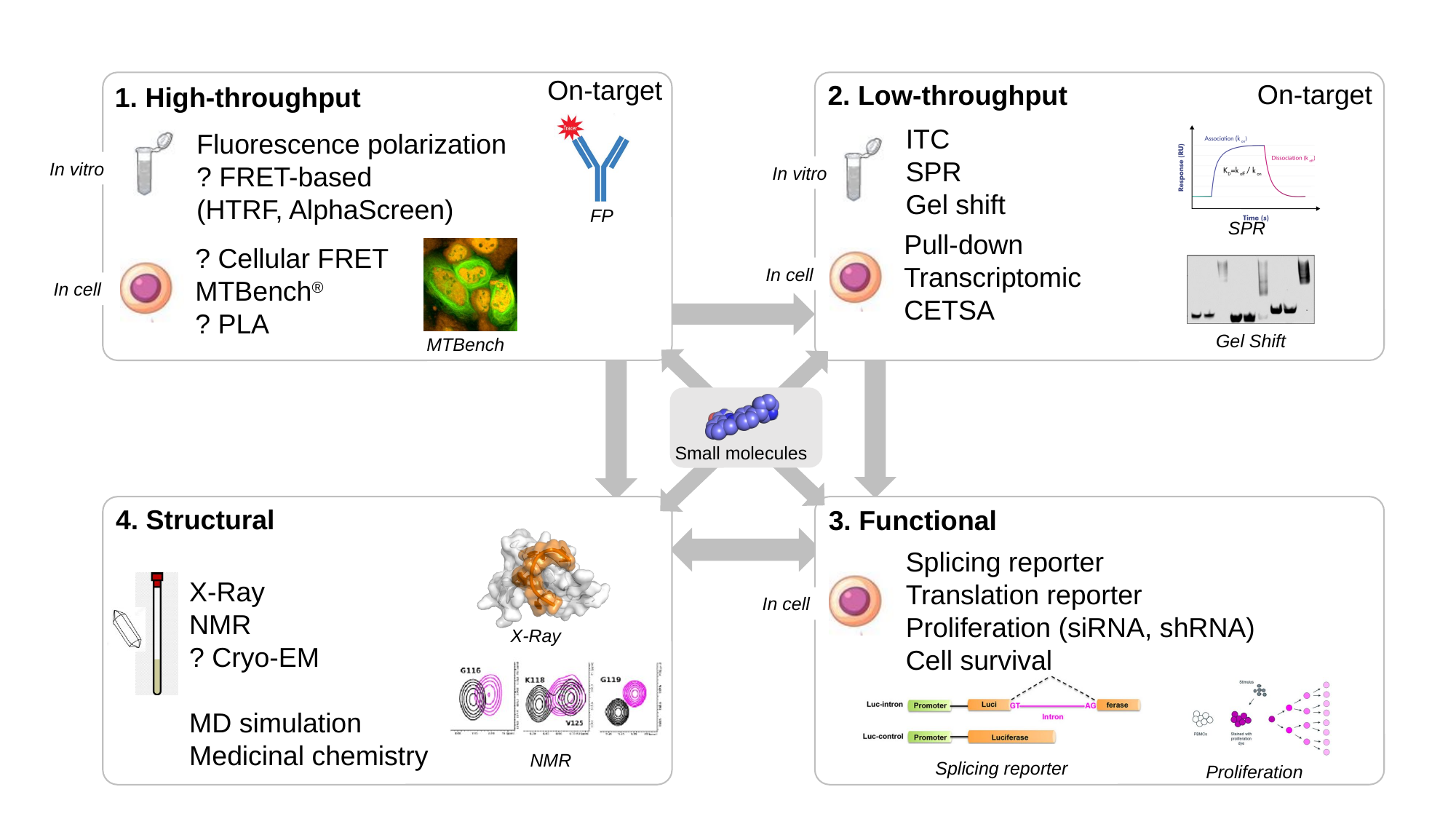

Figure 3.
On-target
On-target
2. Low-throughput
1. High-throughput
ITC
SPR
Gel shift
Fluorescence polarization
? FRET-based
(HTRF, AlphaScreen)
In vitro
In vitro
FP
SPR
Pull-down
Transcriptomic
CETSA
? Cellular FRET
MTBench®
? PLA
In cell
In cell
Gel Shift
MTBench
Small molecules
4. Structural
3. Functional
Splicing reporter
Translation reporter
Proliferation (siRNA, shRNA)
Cell survival
X-Ray
NMR
? Cryo-EM
MD simulation Medicinal chemistry
In cell
X-Ray
NMR
Splicing reporter
Proliferation

## Slide 7
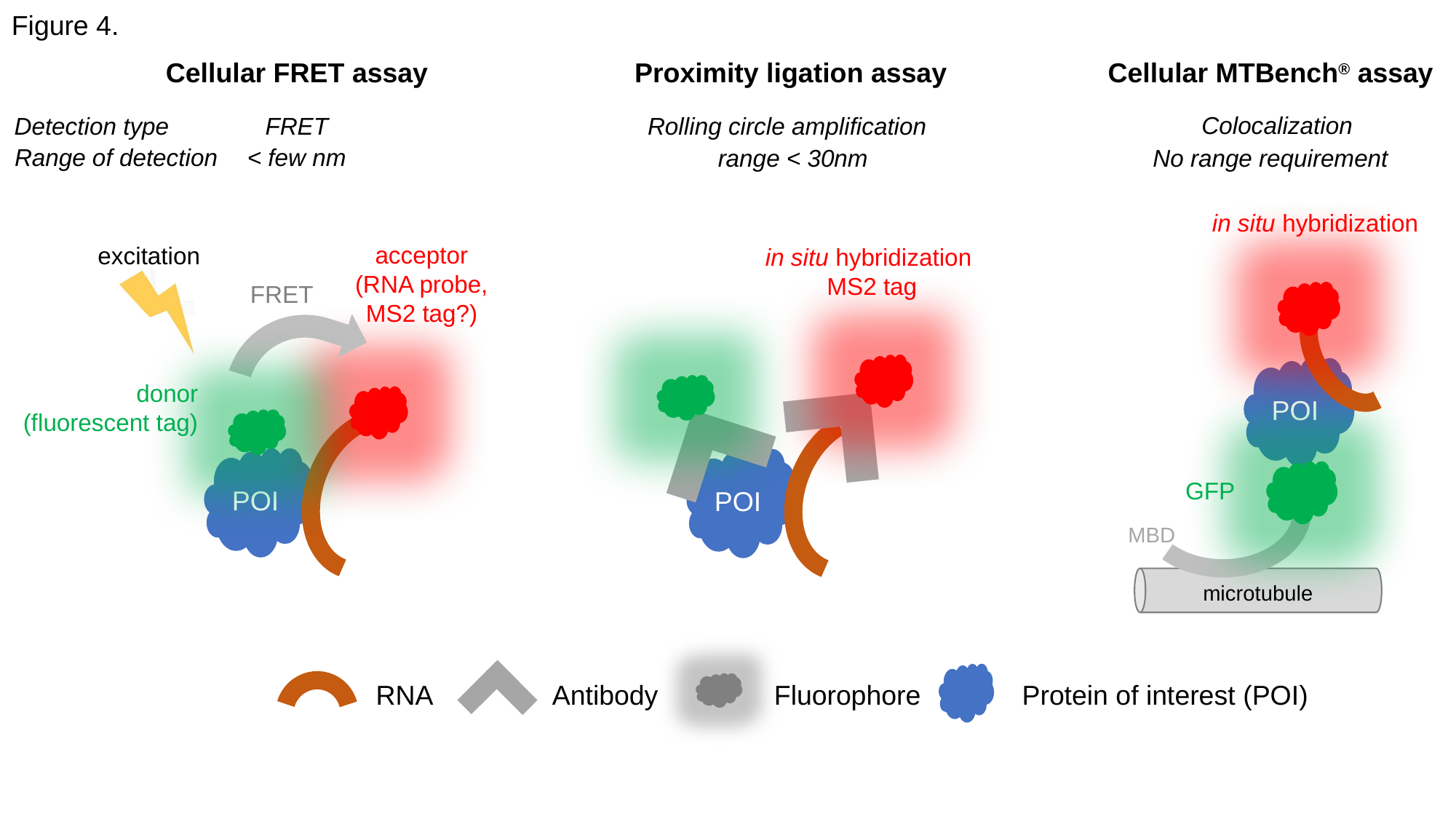

Figure 4.
Cellular FRET assay
Proximity ligation assay
Cellular MTBench® assay
Colocalization
Rolling circle amplification
FRET
Detection type
Range of detection
< few nm
range < 30nm
No range requirement
in situ hybridization
acceptor
(RNA probe, MS2 tag?)
excitation
in situ hybridization
MS2 tag
FRET
POI
donor
(fluorescent tag)
POI
POI
GFP
MBD
microtubule
RNA
Antibody
Fluorophore
Protein of interest (POI)
