# Supplementary material for: Using the structural diversity of RNA: protein interfaces to selectively target RNA with small molecules in cells: methods and perspectives
Source: Front Mol Biosci. 2023 Nov 16;10:1298441. doi: 10.3389/fmolb.2023.1298441 (PMC10687564; doi:10.3389/fmolb.2023.1298441)
Supplement: Supplementary file 2 [file Presentation2.PPTX]

## Slide 1
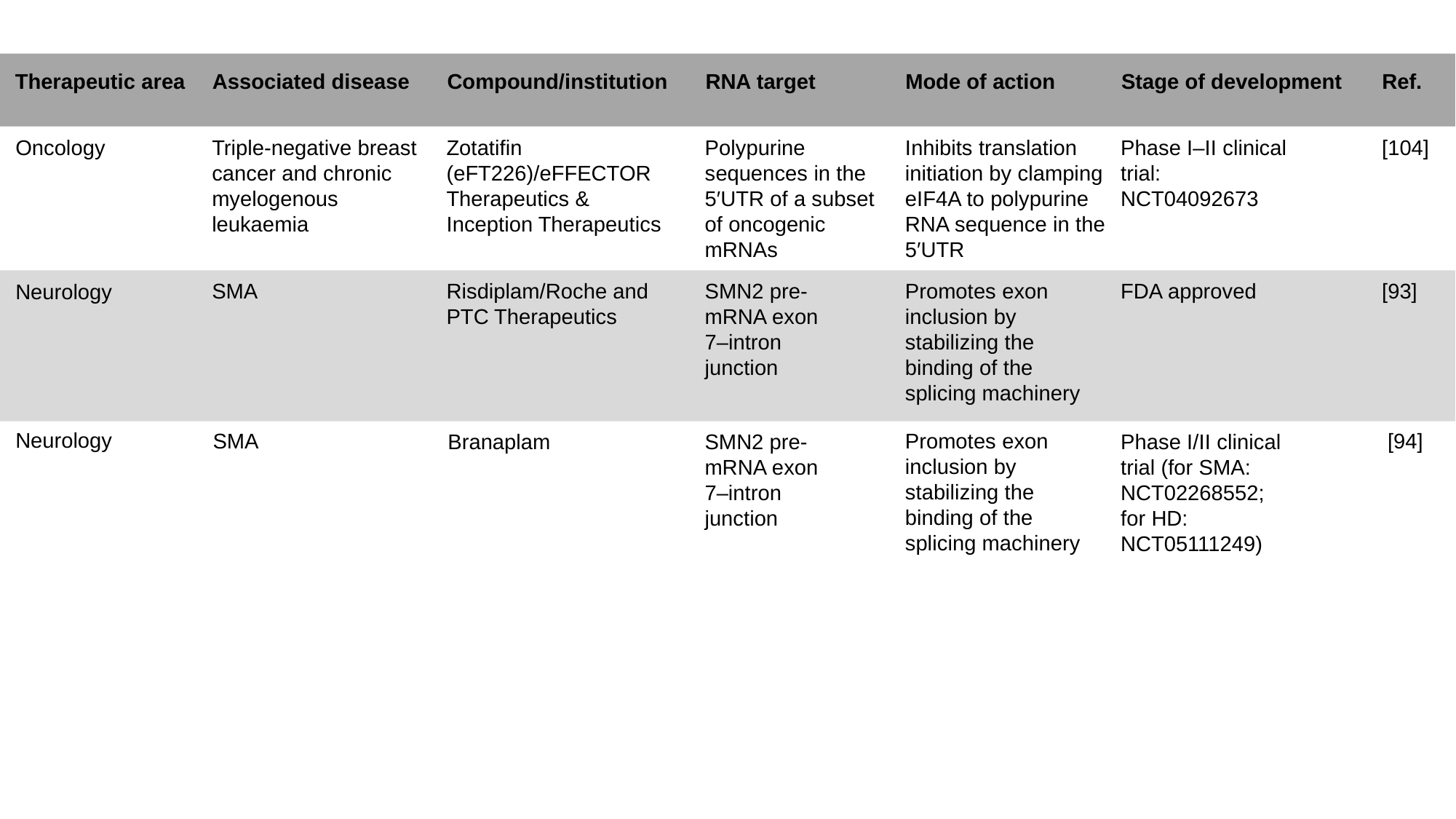

Ref.
Stage of development
Therapeutic area
Associated disease
Compound/institution
RNA target
Mode of action
Inhibits translation initiation by clamping eIF4A to polypurine RNA sequence in the 5′UTR
Phase I–II clinical trial: NCT04092673
[104]
Oncology
Triple-negative breast cancer and chronic myelogenous leukaemia
Zotatifin (eFT226)/eFFECTOR Therapeutics & Inception Therapeutics
Polypurine sequences in the 5′UTR of a subset of oncogenic mRNAs
[93]
Promotes exon inclusion by stabilizing the binding of the splicing machinery
FDA approved
SMA
Risdiplam/Roche and PTC Therapeutics
SMN2 pre-mRNA exon 7–intron junction
Neurology
Neurology
SMA
[94]
Promotes exon inclusion by stabilizing the binding of the splicing machinery
Phase I/II clinical trial (for SMA: NCT02268552; for HD: NCT05111249)
Branaplam
SMN2 pre-mRNA exon 7–intron junction
